# Identification of four novel cytochrome P4501B1 mutations (p.I94X, p.H279D, p.Q340H, and p.K433K) in primary congenital glaucoma patients

**Published:** 2009-12-30

**Authors:** Mukesh Tanwar, Tanuj Dada, Ramanjit Sihota, Rima Dada

**Affiliations:** 1 Laboratory For Molecular Reproduction and Genetics, Department of Anatomy, All India Institute of Medical Sciences, Ansari Nagar, New Delhi, India; 2 Dr. R.P. Centre for Ophthalmic Sciences, All India Institute of Medical Sciences, Ansari Nagar, New Delhi, India

## Abstract

**Purpose:**

Primary congenital glaucoma (PCG) is an autosomal recessive eye disorder that is postulated to result from developmental defects in the anterior eye segment. Mutations in the cytochrome P4501B1 (*CYP1B1*) gene are a predominant cause of congenital glaucoma. In this study we identify *CYP1B1* mutations in PCG patients.

**Methods:**

Twenty-three unrelated PCG patients and 50 healthy controls were enrolled in the study. *CYP1B1* was screened for mutations by PCR and DNA sequencing.

**Results:**

DNA sequencing revealed a total of 15 mutations. Out of these, four (p.I94X, p.H279D, p.Q340H, and p.K433K) were novel mutations and five were known pathogenic mutations. Five coding single nucleotide polymorphisms and one intronic single nucleotide polymorphism (rs2617266) were also found. Truncating mutations (p.I94X and p.R355X) were associated with the most severe disease phenotype. It is possible that patients with two null alleles with no catalytic activity may present with a more severe phenotype of the disease compared to patients with one null allele (heterozygous). The disease phenotype of patients with *CYP1B1* mutations was more severe compared with the clinical phenotype of patients negative for *CYP1B1* mutations.

**Conclusion:**

Mutations in *CYP1B1* are a major cause for PCG in our patients. Identifying mutations in subjects at risk of developing glaucoma, particularly among relatives of PCG patients, is of clinical significance. These developments may help in reducing the disease frequency in familial cases. Such studies will be of benefit in the identification of pathogenic mutations in different populations and will enable us to develop simple and rapid diagnostic tests for analyzing such cases.

## Introduction

Primary congenital glaucoma (PCG; OMIM 231300) is an autosomal recessive disorder of the eye. In this disease the trabecular meshwork (TM) and anterior chamber of the eye are affected, leading to impairment in the aqueous drainage, increased intraocular pressure (IOP), and optic nerve damage. PCG occurs during the neonatal or early infantile period [1]. The term PCG is reserved for those cases in which the only anatomic defect observed is isolated trabeculodysgenesis. This increased IOP results in ocular enlargement (buphthalmos), corneal clouding, and rapid optic nerve cupping. Progressive degeneration of the retinal ganglion cells (RGCs) results in the characteristic optic nerve atrophy and visual field defects found in glaucoma. Most cases of PCG are sporadic, but familial cases have also been reported. PCG is the most common type of pediatric glaucoma and accounts for 55% of such cases. Its expression and penetrance vary from 40–100%. Its incidence varies substantially from one population to another. It is estimated to occur in 1 in 10,000 births in Europe and 1 in 3,300 births in Andhra Pradesh, India [2,3].

Recently a putative PCG locus, GLC3A, was linked to markers on the short arm of chromosome 2 in 11 Turkish families [4]. Six other families did not show linkage to this locus, suggesting locus heterogeneity for this disease. Another PCG locus, GLC3B, was localized on chromosome 1p36 in some families but did not show linkage to chromosome 2 markers [5]. Other subsets of families that did not show linkage to these two loci provide evidence for at least a third of the unmapped loci [5]. Recently Stoilov et al. [6] identified three different mutations in the cytochrome P4501B1 (*CYP1B1*) gene in five unrelated Turkish families in which the disease had been linked to the 2p21 locus [6]. Even though three different loci have been mapped for PCG, mutations in *CYP1B1* (GLC3A) are the most predominant cause of disease and are reported in various ethnic backgrounds [6-15]. Further, it is estimated that all the known loci/genes of glaucoma account for a minority of the total cases of glaucoma [4,5], and hence many other genes remain to be identified.

*CYP1B1* is located on chromosome 2 and consists of three exons and two introns. The coding region of *CYP1B1* starts at the 5′ end of exon 2 and ends within exon 3. It codes for a 543-amino acid protein and is expressed in the ocular tissues, such as the anterior chamber, and in several nonocular tissues [16]. CYP1B1 is a member of the cytochrome P450 superfamily of drug-metabolizing enzymes. It catalyzes several oxidative reactions, some of which are biosynthetic, producing necessary hormones or compounds of intermediary metabolism in most living organisms and substrates, including many xenobiotics, vitamins, and steroids [17]. CYP1B1 also metabolizes vitamin A in two steps to all-trans-retinal and all-trans-retinoic acid. The latter is a potent morphogen and regulates in utero development of tissue growth and differentiation. CYP1B1 is involved in the metabolism of endogenous and exogenous substrates that take part in early ocular differentiation [18-20]. In the present study we screened all coding exons of *CYP1B1* in 23 unrelated congenital glaucoma patients.

## Methods

### Clinical evaluation and patient selection

Primary congenital glaucoma cases presenting at the Dr. R. P. Centre for Ophthalmic Sciences (AIIMS, New Delhi, India), were enrolled for this study. Six patients were female and 17 were male. Mean age of presentation was 15.17 months (range 1.5 – 132 months). After ethical approval of the Institutional Review Board (IRB00006862; All India Institute of Medical Sciences, New Delhi, India), 23 PCG cases were enrolled in this study. The diagnosis involved clinical ocular and systemic examination. Inclusion criteria of the patients were increased corneal diameter (>12.0 mm) and raised IOP (>21 mmHg) with presence/absence of Haab’s striae and optic disc changes (where examination was possible). Symptoms of epiphora and photophobia were the additional inclusion factors. The age of onset ranged from birth to 3 years. Detailed family histories up to three generations were taken, and pedigree charts were constructed. The history of ocular or other hereditary disorders was recorded. Glaucoma cases other than PCG were excluded. Fifty ethnically matched normal individuals without any ocular disorders were enrolled as controls. Peripheral blood samples were collected from patients and controls by venipuncture after informed consent. Blood samples were collected in EDTA vaccutainer and stored in -80 ^°^C until DNA isolation.

### Mutation screening and sequence analysis

Genomic DNA was isolated from peripheral blood by the phenol chloroform method. The entire coding region, including exon–intron boundaries of *CYP1B1*, from patients and controls was amplified and screened for mutations by using three sets of overlapping primers (Table 1) [7,21]. The primers used were set I (1F–1R, 786 bp) [12], set II (2F–2R, 787 bp) [13], and set III (3F–3R, 885 bp) [12]. PCR amplifications for primer sets I and II were performed in a 40 µl volume containing 1.0 µl of 20 µM stock solution for each primer, 100 ng of genomic DNA, 1 unit of Taq polymerase (Banglore Genei), 0.1 mM of each dNTP, 4 µl of 10X PCR buffer (with 15 mM MgCl_2_) and 4 µl of dimethyl sulphoxide (Sigma), by means of 35 cycles of amplification, each consisting of 30 s denaturation at 94 ^°^C, 30 s annealing at 56 ^°^C and 1 min extension at 72 ^°^C [12], while conditions for set III were initial denaturation at 94 °C for 3 min followed by 30 cycles each at 94 °C for 30 s, 60 °C for 30 s, and 72 °C for 1 min.

**Table 1:** The primers used for PCR amplification.

**Primer sequence**	**Product size (bp)**
1F-5′-TCTCCAGAGAGTCAGCTCCG-3′	786
1R-5′-GGGTCGTCGTGGCTGTAG-3′	
2F-5′-ATGGCTTTCGGCCACTACT-3′	787
2R-5′-GATCTTGGTTTTGAGGGGTG-3′	
3F-5′-TCCCAGAAATATTAATTTAGTCACTG-3′	885
3R-5′-TATGGAGCACACCTCACCTG-3′	

Amplified PCR products were purified using a gel/PCR DNA fragments extraction kit (number DF100; Geneaid Biotech Ltd., Sijhih City, Taiwan). Purified PCR products were sent for sequencing to MCLAB (Molecular Cloning Laboratories, South San Francisco, CA). DNA sequences were analyzed against the *CYP1B1* reference sequence ENSG00000138061 using ClustalW2 (multiple sequence alignment program for DNA; European Molecular Biology Laboratory (EMBL) – European Bioinformatics Institute (EBI)).

### Computational assessment of missense mutations

Two homology-based programs PolyPhen (polymorphism phenotyping; Division of Genetics, Department of Medicine, Brigham and Women’s Hospital/Harvard Medical School, Boston, MA) and SIFT (sorting intolerant from tolerant; the J. Craig Venter Institute Rockville, MD and La Jolla, CA) were used to predict the functional impact of missense changes identified in this study. PolyPhen structurally analyzes an amino acid polymorphism and predicts whether that amino acid change is likely to be deleterious to protein function [22-24]. The prediction is based on the position-specific independent counts (PSIC) score derived from multiple sequence alignments of observations. PolyPhen scores of >2.0 indicate the polymorphism is probably damaging to protein function. Scores of 1.5–2.0 are possibly damaging, and scores of <1.5 are likely benign. SIFT is a sequence homology-based tool that sorts intolerant from tolerant amino acid substitutions and predicts whether an amino acid substitution in a protein will have a phenotypic effect [25-28]. SIFT is based on the premise that protein evolution is correlated with protein function. Positions important for function should be conserved in an alignment of the protein family, whereas unimportant positions should appear diverse in an alignment. Positions with normalized probabilities <0.05 are predicted to be deleterious and those ≥0.05 are predicted to be tolerated.

## Results

All cases were found to be sporadic in origin. A total of 15 nucleotide changes were observed in this study. Out of these, five were previously reported coding single nucleotide polymorphisms (SNPs) and one was already reported as an intronic SNP; five were known pathogenic *CYP1B1* mutations. Four novel nucleotide changes (two nonsynonymous, one frameshift, and one synonymous mutation) were also found in this study. Details of all nucleotide changes are presented below.

### Identification of four novel mutations

#### Isoleucine94stop (p.I94X) mutation

**Figure 1:**
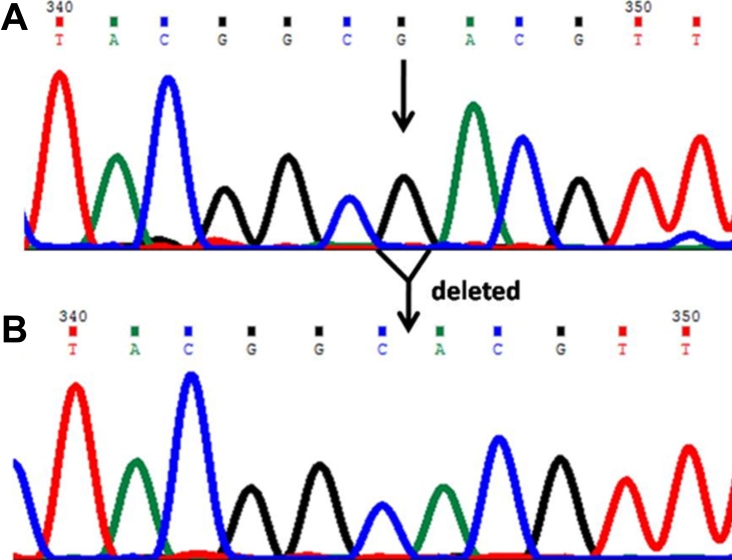
DNA sequence chromatogram of *CYP1B1* exon 2 equivalent to codon 81–85. **A**: The reference sequence derived from control is shown. **B**: Sequence derived from congenital glaucoma patient P55 shows the homozygous deletion of G at c.247, which caused a p.asp83thrfsX12 (p.I94X) mutation.

In this mutation a single base guanine (G) deletion (Figure 1) was observed at genomic position 38302285, coding nucleotide number c.247. This caused a frameshift after codon 82 and introduced a stop codon (TAG) at position 94 in the protein. This mutation produced a truncated CYP1B1 protein of 93 amino acids. This change was identified as a homozygous mutation in the patient (P55).

#### Histidine279aspartic acid (p.H279D) mutation

**Figure 2:**
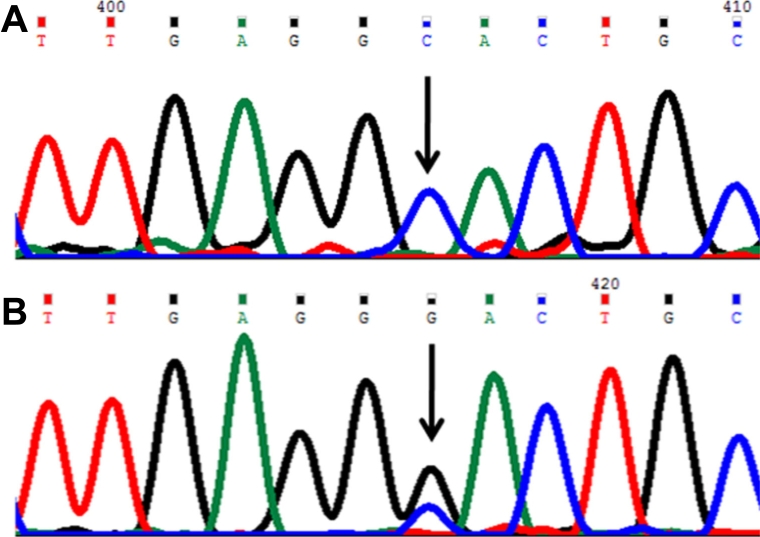
DNA sequence chromatogram of *CYP1B1* exon 2 equivalent to codon 277–280. ** A**: The reference sequence derived from control is shown. **B**: Sequence derived from congenital glaucoma patient P55 shows heterozygous c.835C>G, which predicts a codon change of CAC>GAC and a p.H279D mutation.

In this mutation a single base cytosine (C) was replaced by G (Figure 2) at genomic position 38301697, coding nucleotide number c.835. This resulted in a codon change from CAC to GAC and an amino acid change from histidine to aspartic acid (p.H279D), a nonsynonymous mutation in the CYP1B1 protein. This mutation was identified in one patient (P61) and was heterozygous.

#### Glutamine340histidine (p.Q340H) mutation

**Figure 3:**
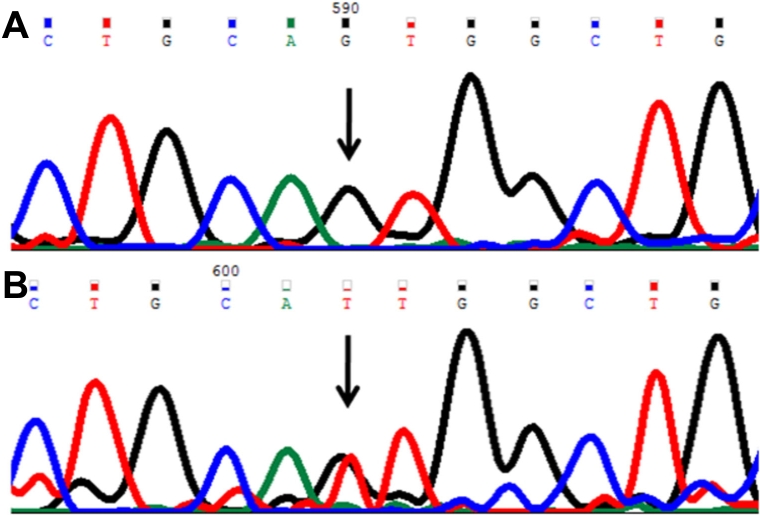
DNA sequence chromatogram of *CYP1B1* exon 2 equivalent to codon 339-342. **A**: The reference sequence derived from control is shown. **B**: Sequence derived from congenital glaucoma patient P56 shows heterozygous c.1020G>T change, which predicts a codon change CAG>CAT and heterozygous p.Q340H mutation.

In this mutation a single base G was replaced by thymine (T) (Figure 3) at genomic position 38301512, coding nucleotide number c.1020. This resulted in a codon change from CAG to CAT and an amino acid change from glutamine to histidine (p.Q340H), a nonsynonymous mutation in the CYP1B1 protein. This mutation was identified in one patient (P56) and was heterozygous and present with the p.R390H mutation in this patient.

#### Lysine433lysine (p.K433K) mutation

**Figure 4:**
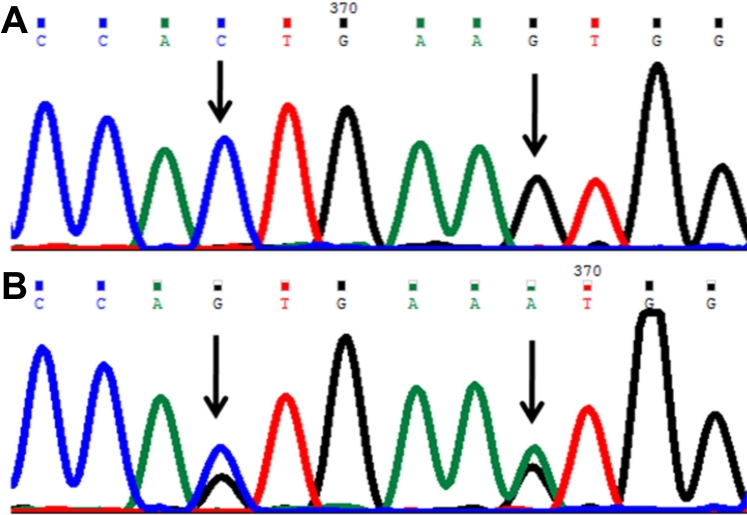
DNA sequence chromatogram of *CYP1B1* exon 3 equivalent to codon 431-434. **A**: The reference sequence derived from control is shown. **B**: Sequence derived from congenital glaucoma patient P69 shows heterozygous c.1294C>G and heterozygous c.1299G>A, which predicts codon change CTG>GTG and AAG>AAA and heterozygous p.L432V and p.K433K mutations, respectively.

In this mutation a single base G was replaced with adenine (A) (Figure 4) at genomic position 38298198, coding nucleotide number c.1299. This resulted in a codon change from AAG to AAA and resulted in no amino acid change (lysine). This was a neutral mutation (p.K433K) in patient P69.

All four novel mutations p.I94X, p.H279D, p.Q340H, and p.K433K have been registered in GenBank with accession numbers GQ925803, GQ925804, GQ925805, and GQ925806, respectively.

### Other previously reported pathogenic *CYP1B1* mutations

#### Glutamic acid229lysine (p.E229K) mutation

This mutation resulted in G being replaced with A at genomic position 38301847 (rs57865060), coding nucleotide number c.685. This resulted in a codon change from GAA to AAA and an amino acid change from glutamic acid to lysine (p.E229K), a nonsynonymous mutation in the CYP1B1 protein. This change was found in one patient (P65) and was heterozygous.

#### Arginine355stop (p.A355X) mutation

**Figure 5:**
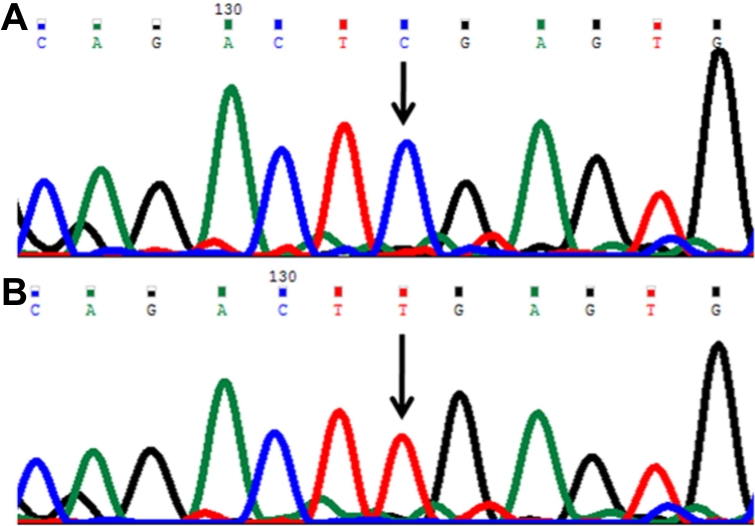
DNA sequence chromatogram of *CYP1B1* exon 3 equivalent to codon 353-356. ** A**: The reference sequence derived from control is shown. **B**: Sequence derived from congenital glaucoma patient P70 shows homozygous c.1063C>T, which predicts a codon change CGA>TGA and p.R355X nonsense mutation.

In this mutation a single base C was replaced by T (Figure 5) at genomic position 38298434, coding nucleotide number c.1063. This resulted in a codon change from CGA to TGA (p.R355X), a nonsense mutation in the CYP1B1 protein. This resulted in a truncated CYP1B1 protein of 355 amino acids. The p.R355X mutation was described only once in the literature [29]. This change was homozygous in one patient (P70).

#### Arginine368histidine (p.R368H) mutation

In this mutation a single base G was replaced by A at genomic position 38298394 (rs28936414), coding nucleotide number c.1103. This resulted in a codon change from CGT to CAT and an amino acid change from arginine to histidine (p.R368H), a nonsynonymous mutation. This change was homozygous in one patient (P68).

#### Arginine390cysteine (p.R390C) mutation

In this mutation a single base C was replaced by T at genomic position 38298329 (rs56010818), coding nucleotide number c.1168. This resulted in a codon change from CGC to TGC and an amino acid change from arginine to cysteine (p.R390C), a nonsynonymous mutation. This mutation was identified in one patient (P64) and was heterozygous.

#### Arginine390histidine (p.R390H) mutation

**Figure 6:**
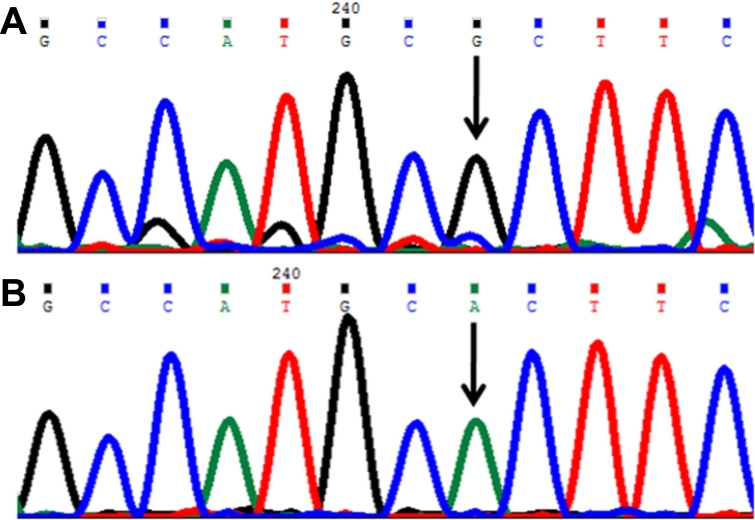
DNA sequence chromatogram of *CYP1B1* exon 3 equivalent to codon 388-391. **A**: The reference sequence derived from control is shown. **B**: Sequence derived from congenital glaucoma patient P56 shows homozygous c.11169G>A, which predicts a codon change CGC>CAC and p.R390H mutation.

In this mutation a single base G was replaced by A (Figure 6) at genomic position 38298328, coding nucleotide number c.1169. This resulted in a codon change from CGC to CAC and an amino acid change from arginine to histidine (p.R390H), a nonsynonymous mutation. This mutation was identified in one patient (P56) and was heterozygous.

### Nonpathogenic *CYP1B1* single nucleotide polymorphisms

In addition to these pathogenic mutations, six previously reported single nucleotide polymorphisms [8] were identified in a less conserved region of the CYP1B1 protein. Details of these polymorphisms are provided below.

#### Cytisine (C) to thymine (T) change in intron 1

In this mutation, C was replaced by T at genomic position 38302544, nucleotide position 780 in *CYP1B1* (rs2617266) in intron I. This was observed in 13 patients but was absent in controls.

#### Arginine48glycine (p.R48G)

In this mutation, C was replaced by guanine (G) at genomic position 38302390 (rs10012), coding nucleotide number c.142. This resulted in a codon change from CGG to GGG and an amino acid change from arginine to glycine (p.R48G), a nonsynonymous mutation in the CYP1B1 protein. This change was also present in controls.

#### Alanine119serine (p.A119S)

In this mutation G was replaced by T at genomic position 38302177 (rs1056827), coding nucleotide number c.355. This resulted in a codon change from GCC to TCC and an amino acid change from alanine to lysine (p.A119S), a nonsynonymous mutation in the CYP1B1 protein. This change was found in patients P55 and P73 but absent in controls.

#### Leucine432valine (p.L432V)

In this mutation a single base C was replaced by G at genomic position 38298203 (rs1056836), coding nucleotide number c.1294. This resulted in a codon change from CTG to GTG and an amino acid change from leucine to valine (p.L432V), a nonsynonymous mutation in the CYP1B1 protein. This mutation was identified in four patients; it was homozygous in three patients (P52, P55, and P68) and heterozygous in one patient (P69) and was also present in controls.

#### Aspartic acid449aspartic acid (p.D449D)

In this mutation a single base T was replaced by C at genomic position 38298150 (rs1056837), nucleotide position 5174 in the gene, coding nucleotide number c.1347. This resulted in a codon change from GAT to GAC and no change in the amino acid (aspartic acid) (p.D449D), a synonymous mutation in the CYP1B1 protein. This mutation was identified in 20 patients and was homozygous in all. This change was also present in controls.

#### Asparagine453serine (p.N453S)

In this mutation a single base A was replaced by G at genomic position 38298139 (rs1800440), coding nucleotide number c.1358. This resulted in a codon change from AAC to AGC and an amino acid change from asparagine to serine (p.N453S), a nonsynonymous mutation in the CYP1B1 protein. The p.N453S mutation was present in two patients (P51 and P62) but absent in controls.

The clinical manifestations of PCG patients have been tabulated (Table 2), and the *CYP1B*1 sequence variants identified in the various studies to date have been summarized (Table 3). The clinical phenotype of the cases with pathogenic *CYP1B1* mutations was more severe compared to cases without *CYP1B1* mutations. The mean IOP of cases with pathogenic *CYP1B1* mutations was 30.21 mmHg compared to 23.96 mmHg in mutation-negative cases; the difference is significant (p value <0.005). The mean corneal diameter in patients without the *CYP1B1* mutations was 12.625×12.181 mm (left eye) and 12.406×12.781 mm (right eye), whereas it was 13.833×13.750 mm (left eye) and 13.416×15.50 mm (right eye) in mutation-negative cases. Haab’s striae were present in two cases (P56 and P61), which were positive for the *CYP1B1* mutations.

**Table 2:** Clinical manifestations of PCG patients.

**Pt. ID**	**Age of onset of disease**	**Sex**	**Age at presentation/sampling**	**Corneal Diameter (mm) OS/OD and clarity at diagnosis**	**Buphthalmos**	**IOP OS/OD (mmHg) At presentation**	**Haabs’ striae**	**Last Cup Disc ratio OS/OD**	**Photo-phobia**	**Mutations**	**Treatments**
P51	By birth	F	36 months	11x11.5/13x13; OU mild edema	OU;OD>OS	22/28	no	0.8:1/0.9:1	Yes	—	Medical and OU Trab/Trab+MMC; OU cataract surgery
P52	By Birth	F	2 months	12.5x13/12.5x13; OS mild edema	OU	20/20	no	Hazy media/0.5:1	Yes	—	Medical and OU Trab/Trab+MMC
P53	By birth	F	4 months	11.5x12/12x12; OU mild edema	OU; OD>OS	40/23	no	Hazy media	Yes	—	Medical and OU Trab/Trab+MMC
P54	By birth	M	9 months	15x14.5/15x14.5; no edema	OU	26/26	no	No glow	No	—	Medical and OUTrab/Trab+MMC
P55	By birth	M	8 months	Phthisic eye/12x12; OU severe edema	OD; OS Phthisic eye	NA/37	no	NA/0.9:1	No	p.I94X (H)	Medical and OD Trab/Trab+MMC
P56	By birth	F	12 months	14.5x14.5/14x14; OU severe edema	OU; OS>OD	30/28	OU +ve	Hazy media	No	p.Q340H (H) + p.R390H (H)	Medical and OU Trab/Trab+MMC
P57	By birth	M	3 months	13x13/13.5x13.5; No edema	OU; OD>OS	28/30	no	0.7:1/0.7:1	No	—	Medical and OU Trab/Trab+MMC
P58	By birth	M	15 months	14x14/12.5x12.5; No edema	OU; OS>OD	20/16	no	total cupping/ 0.5:1	No	—	Medical and OU Trab/Trab+MMC
P59	By birth	F	10 months	14x14.5/13.5x14; OS edema	OU; OS>OD	31/31	OS +ve	Not available	No	—	Medical and OU Trab/Trab+MMC
P60	7 months	F	41 months	14x14/13x13.5; no edema	OU; OS>OD	24/18	no	0.4:1/0.6:1	Yes	—	Medical and OU Trab/Trab+MMC
P61	By birth	M	4 months	15.5x15/14x14; OU mild edema	OU; OS>OD	30/34	OU +ve	Not visible/0.9:1	No	p.H279D (h)	Medical and OU Trab/Trab+MMC
P62	By birth	M	8 months	11x11.5/10x11.5; OD edema	OU; OS>OD	22/16	no	0.6:1/0.6:1	Yes	—	Medical and OU Trab/Trab+MMC
P63	3 months	M	12 months	12x12/11x12.5; No edema	OU; OS>OD	22/23	no	0.4:1/0.4:1	No	—	Medical and OU Trab/Trab+MMC
P64	By birth	M	1 month	12x12/11.5x11.5; OD severe edema	OU; OS>OD	28/24	no	Not visible/0.7:1	Yes	p.R390C (h)	Medical and OU Trab/Trab+MMC
P65	By birth	M	6 months	13x13/12.5x13; OU edema	OU; OS>OD	25/26	no	0.7:1/0.7:1	No	p.E229K (h)	Medical and OU Trab/Trab+MMC
P66	3 months	M	13 months	12.5x12/12.5x12; No edema	OU	22/24	no	0.4:1/0.4:1	no	—	Medical and OU Trab/Trab+MMC
P67	11 months	M	132 months	12x13/13x14; OU mild edema	OU; OD>OS	21/24	no	Not visible	Yes	—	Medical and OU Trab/Trab+MMC
P68	By birth	M	6 months	13x13/13.5x14; OU severe edema	OU; OD>OS	26/28	no	0.8:1/0.8:1	no	p.R368H (H)	Medical and OU Trab/Trab+MMC
P69	By birth	M	4 months	13x13/12x13; No edema	OU; OS>OD	24/26	no	0.5:1/0.6:1	no	—	Medical and OU Trab/Trab+MMC
P70	By birth	M	45 days	15x15/15x14.5; OU severe edema	OU/; OS>OD	30/40	Not visible	Not visible	no	p.R355X (H)	Medical and OU Trab/Trab+MMC
P71	13 months	M	18 months	12x2/12x12; No edema	OU	22/23	no	0.4:1/0.4:1	Yes	—	Medical and OU Trab/Trab+MMC
P72	By birth	M	45 days	12x12/12x11.5; OU mild edema	OU; OS>OD	20/24	no	No glow	Yes	—	Medical and OU Trab/Trab+MMC
P73	By month	M	2 months	12.5x13/11x12; OU mild edema	OU; OS>OD	22/22	no	Hazy media	no	—	Medical and OU Trab/Trab+MMC

Footnote: M- male; F- female; H-homozygous; h-heterozygous; X- times; Trab/Trab+MMC- combined trabeculotomy trabeculectomy and mitomycin C treatment; OD- right eye; OS- left eye; OU- both eyes; mutations in bold letters- novel mutations.

**Table 3:** Summary of the sequence variants identified in various studies till date.

**S. No.**	**Patient number**	**Genomic location**	**Nucleotide change**	**Codon change**	**Type of mutation**	**Location in protein**	**Mutation identified**	**Observational history of mutations in different diseases**	**Origin (reference)**
1	P51, P53, P55, P58-P65, P71, P72, P73	g.38302544	C>T	NA	Intronic	NA	NA	PCG	India, Saudia Arabia, Oman, Brazil [7,13,35,36]
2	P51, P53, P54, P57-P61, P63-P65, P71-P73	g.38302390	c.142 C>G	CGG>GGG	Missense	48	p.arg48 gly (p.R48G)	PCG	Saudia Arabia, India, Japan [8,11,15]
3	P55	g.38302285	c.247del. G	FS	FS	FS after 82	p.asp83thrfsX12 (p.I94X)	PCG	This study
4	P55, P73	g.38302177	c.355G>T	GCC>TCC	Missense	119	p.ala119ser (p.A119S)	PCG	Saudia Arabia, Japan, India [7,11,15]
5	P65	g.38301847	c.685G> A	GAA>AAA	Missense	229	p.glu229lys (p.E229K)	PCG, POAG	France, India, Germany [7,29,31]
6	P61	g.38301697	c.835C>G	CAC>GAC	Missense	279	p.his279asp (p.H279D)	PCG	This study
7	P56	g.38301512	c.1020G>T	CAG>CAT	Missense	340	p.gln340his (p.Q340H)	PCG	This study
8	P70	g.38298434	c.1063C>T	GGA>TGA	Non-sense	355	p.ala355stop (p.A355X)	PCG	Germany, India [28] this study
9	P68	g.38298394	c.1103G>A	CGT>CAT	Missense	368	p.arg368his (p.R368H)	PCG, PA, POAG	Saudia Arabia, India. France [7,15,32]
10	P64	g.39298329	c.1168C>T	CGC>TGC	Missense	390	p.arg390cys (p.R390C)	PCG	Ecuador, India [8,37]
11	P56	g.38298328	c.1169G>A	CGC>CAC	Missense	390	p.arg390his (p.R390H)	PCG, POAG	Pakistan, India, France [7,21,32]
12	P52, P55, P68, P69	g.38298203	c.1294C>G	CTG>GTG	Missense	432	p.leu432lys (p.L432V)	PCG, PA	India, Japan, Turkey [7,11,29]
13	P69	g.38298198	c.1299G>A	AAG>AAA	Neutral	433	p.lys433lys (p.K433K)	PCG	This study
14	P51, P53-P67, P69-P71, P73	g.38298350	c.1347T>C	GAT>GAC	Neutral	449	p.asp449asp (p.D449D)	PCG	Japan, India [7,11]
15	P56, P67	g.38298139	c.1358A>G	AAC>AGC	Missense	453	p.asp453ser (p.N453S)	PCG	France, India [7,12]

Footnote: PCG- Primary congenital glaucoma; POAG- Primary open angle glaucoma; PA-Peter’s anomaly; FS-frameshift; X- stop codon; NA- not applicable; mutations in bold letters- novel mutations.

## Discussion

### Structural/functional implications of mutations

#### p.I94X mutation

**Figure 7:**
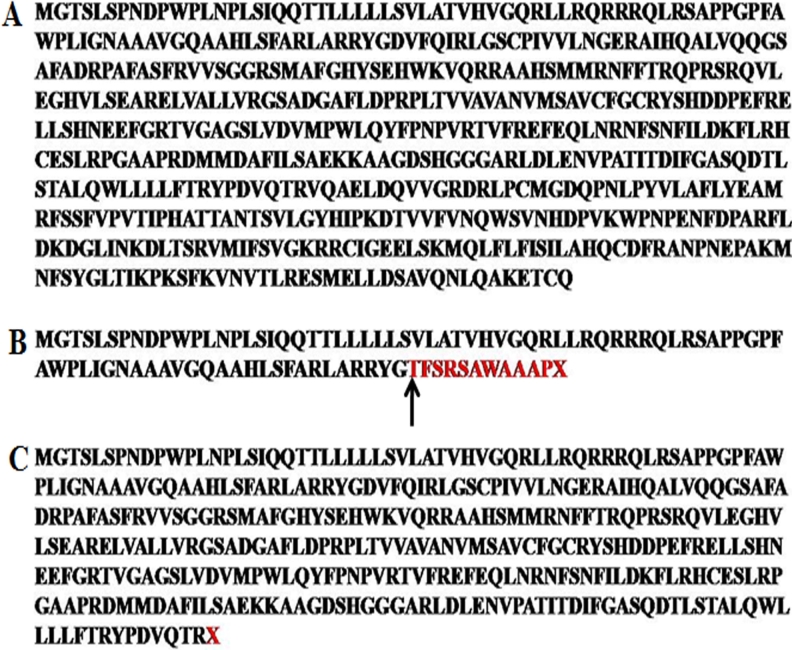
Amino acid sequence of CYP1B1 protein.** A**: Wild-type CYP1B1 protein. **B**: Truncated CYP1B1 protein of 93 amino acids (black arrow shows the position after which frameshift takes place and red letters shows amino acids after frameshift). **C**: Truncated CYP1B1 protein of 354 amino acids.

In the isoleucine94stop mutation (p.194X) mutation a truncated protein of 93 amino acids is produced in which only the first 82 amino acids are the same as the wild-type CYP1B1 protein (Figure 7). This truncated protein lacks all functional domains of the CYP1B1 protein and is a nonfunctional protein [6,21,29,30].

#### p.H279D mutation

**Figure 8:**
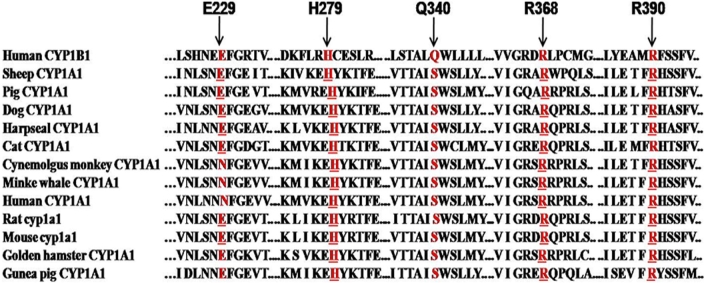
Multi sequence alignment of the human CYP1B1 protein with the CYP1A1 protein from different species. Red Underlined amino acids shows the conserved residues in human CYP1B1 and different CYP1A1 protein from different species (when mutated) causing primary congenital glaucoma phenotype. While Red letter shows amino acid conserved in different CYP1A1 protein from different species but not present in human CYP1B1 protein.

**Figure 9:**
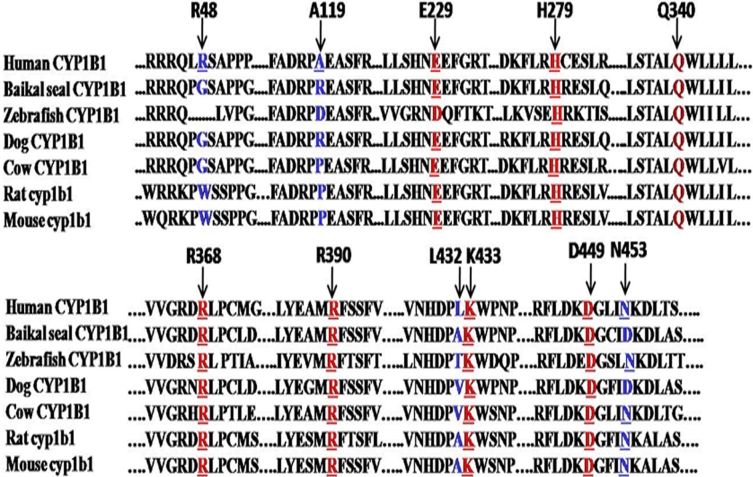
Multisequence alignment of the human CYP1B1 protein with the CYP1B1 protein from different species. Underlined red amino acids show the conserved residues (when mutated) causing the primary congenital glaucoma phenotype. Red colored amino acid shows the non-conservation of glutamic acid at this locus in Zebrafish CYP1B1. Blue-colored amino acids show the less conserved residues in CYP1B1 protein from different species.

This histidine residue lies in the carboxyl terminal of the G helix in the CYP1B1 protein. Replacement of an aromatic, weak basic, amino acid histidine whose charge state depends upon its protonation state with an aliphatic, strong acidic, and negatively charged aspartic acid at this locus. This in turn affects the local charge distribution, and hence the structure of the protein is disturbed. Histidine is conserved at this locus in the CYP1A1 protein from 12 different species (Figure 8) and in the CYP1B1 protein from seven different species (Figure 9) analyzed, suggesting that histidine performs some important functions at this locus. No other known pathogenic mutation was present in the patient (P61), and the PSIC score of this mutation was 2.628, indicating that this change is probably damaging to the protein function. The SIFT score of p.H279D was 0.00 and is predicted to be deleterious for the protein function.

#### p.Q340H mutation

This glutamine residue lies in the carboxyl terminal of the I helix. Replacement of a polar uncharged amino acid (glutamine) with a weak basic amino acid (histidine) may or may not alter the structure/function of the protein. Glutamine is not conserved at this locus in the CYP1A1 protein from 12 different species analyzed (Figure 8) but is conserved in the CYP1B1 protein from seven different species analyzed (Figure 9). The PSIC score of this mutation was 0.276, indicating that this change is benign to protein function. The SIFT score of p.Q340H was 0.05 and is predicted to be tolerated. The patient with the p.Q340H mutation also had a known pathogenic CYP1B1 mutation (p.R390H) and had a PSIC score of 2.799 and a SIFT score of 0.00. The p.R390H mutation has previously been reported [21] to adversely affect or damage protein function.

#### p.E229K mutation

The p.E229k mutation occurred in the carboxyl terminal of the F helix in the vicinity of the substrate-binding region in the CYP1B1 protein. Substitution of E to K leads to a change from a negatively charged residue to a positively charged side chain, and this in turn affects the local charge distribution. This disturbs an important cluster of salt bridges. In wild-type CYP1B1 protein, R-194::E-229, R-194::D-333, and D-333::K-512 form a triangle of ionic bond interactions, holding the I helix with the F helix and β-strand S3.2. As a result of this mutation, the R-194::E-229 interaction is lost, which has the potential to destabilize the other ionic interactions in the protein [30]. The SIFT score of the p.E229K mutation was 0.01 and is predicted to be deleterious for the protein function. The CYP1B1 protein with the p.E229K mutation shows 20–40% enzymatic activity compared to the wild-type CYP1B1 protein [31].

#### p.R355X mutation

In the p.R355X mutation, a truncated protein of 354 amino acids is produced (Figure 7). The arginine residue at position 355 lies in the carboxyl terminal of the J helix, carboyxl terminal of the J helix is involved in the functionally important heme-binding domain. This truncating mutation results in a loss of the heme-binding domain and a functionally inactive protein [6,21,29,30].

#### p.R368H mutation

This arginine residue lies between the J and K helix in an exposed loop [8,15]. In this mutation the positively charged amino acid arginine is replaced by histidine whose charge state depends upon its protonation state. Consequences of this change are not immediately apparent. In the wild type, arginine at position 368 interacts with G-365, D-367, V-363, and D-374. Because of the R368H mutation, interaction between D-367 and D-374 are weakened. The PSIC score of this mutation was 2.653, indicating that this change is probably damaging to protein function. The SIFT score of p.R368H was 0.00 and is predicted to be deleterious for the protein function. How p.R368H affects the conformation and functionality of the protein is still not clear [31].

#### p.R390H/C mutation

This arginine residue is located in the conserved α helix K [8]. It forms the consensus sequence GluXXArg, which is conserved among all members of the cytochrome P450 superfamily [21]. Arg390 and Glu387 are one helical turn apart and are predicted to form a salt bridge. The parallel orientation of their side chains is more transparent in the three-dimensional model. Conservation of this motif indicates that presence of arginine at this position is essential for the normal function of the P450 molecule. The PSIC scores of p.R390C and p.R390H were 3.474 and 2.799, respectively, indicating that both these changes are probably damaging to protein function. The SIFT score of p.R390H/C was 0.00 and is predicted to be deleterious for the protein function.

The PSIC scores of the nonpathogenic single nucleotide polymorphisms were <2 for p.R48G, p.A119S, p.N453S, and p.L432V, indicating that all these changes were benign to protein function. The SIFT scores of the nonpathogenic single nucleotide polymorphisms were >0.05 for p.R48G, p.L432V, p.K433K, and p.D449D, indicating that all these changes were tolerated in the protein.

PCG is a clinically and genetically heterogeneous disorder. More than 50 different mutations have been reported in the entire coding region of *CYP1B1* from various populations. We screened the entire coding region of *CYP1B1* in 23 congenital glaucoma patients by using primers described elsewhere [8]. Of all mutations identified herein, the frameshift mutation (c.247delG) and nonsense mutation (c.1063C>T) resulted in the most severe disease phenotype.

The patient (P55) with the p.I94X (homozygous) mutation is a male child of a consanguineous marriage without any family history of glaucoma; he presented at 8 months of age. He was born at full term through a normal vaginal delivery. He had severe bilateral corneal edema at birth. At the age of 2 months he had congestion with discharge in the left eye and was diagnosed to have a left corneal ulcer and was treated with antibiotics; the left eye consequently developed phthisis. The right eye dimensions increased, and he was diagnosed as having buphthalmos at the age of 8 months. Combined trabeculotomy and trabeculectomy with mitomycin C was performed in his right eye. He was diagnosed as having 100% blindness at 8 months. His parents were also screened for *CYP1B1* mutations by DNA sequencing but were found to be negative for any pathogenic *CYP1B1* mutations. 

Patient P70 has a p.R355X (homozygous) mutation and is a male offspring of a non-consanguineous marriage; he presented at 45 days. At birth, he had bilateral congenital glaucoma and had IOPs of 30 and 40 mmHg in his left and right eye, respectively. He had severe corneal clouding in both eyes, at birth, and therefore the fundus was not visualized. Combined trabeculotomy and trabeculectomy with mitomycin C was performed in both eyes. He had no light perception and was visually blind since 45 days of age. His parents were also negative for the pathogenic *CYP1B1* mutations. The absence of mutations in the parents of P55 and P70 could be due to a parental germline mutation, which cannot be tested by using peripheral leukocytes.

Patient P56 is a female child of a non-consanguineous marriage; she presented at the age of 1 year. She has p.Q340H (heterozygous) and p. R390H (homozygous) mutations. She had bilateral congenital glaucoma since birth. She had a corneal diameter of 14.0×14.0 mm (right eye) and 14.5×14.5 mm (left eye) and IOPs of 28 and 30 mmHg in the right and left eye, respectively. She had Haab’s striae in both eyes, and the fundus was not visible. Combined trabeculotomy and trabeculectomy with mitomycin C was performed in both eyes.

Patient P61 is a male child of a non-consanguineous marriage; he presented at the age of 4 months and has a p.H279D (heterozygous) mutation. He had bilateral congenital glaucoma at birth. At presentation corneal diameter and IOPs of his left and right eye were 15.5×15.0 mm and 14.0×14.0 mm and 30 mmHg and 34 mmHg, respectively. The cup to disc ratio of the left eye was not visible due to the hazy media and that of the right eye was 0.9:1. He had Haab’s striae in both eyes. Combined trabeculotomy and trabeculectomy with mitomycin C was performed in both eyes.

An intriguing finding that apparently does not match a typical recessive pattern of inheritance is the presence of a heterozygous *CYP1B1* mutation in PCG patients. This situation has been previously reported [7,29]. A heterozygous p.Y81N mutation has also been described in PCG patients from Germany, and a heterozygous p.E229K mutation has been identified in unrelated French and Indian patients [7,32]. Few heterozygous *CYP1B1* mutations were associated with the milder, primary, open-angle glaucoma phenotypes in patients from Spain, France, and India. The presence of a heterozygous *CYP1B1* mutation in PCG suggests the possibility of other loci, yet undetected, that may be involved in anterior chamber formation. Recently the presence of double heterozygote variants *CYP1B1* and *FOXC1* has been described in two PCG cases, although the role of possible digenic inheritance in disease causation is yet to be established [33]. Defective variants of modifier genes and/or environmental factors have an additive effect with loss-of-function *CYP1B1* alleles to produce the disease phenotype. However further work is required to understand this mechanism.

Previous studies have reported that the age of disease onset in PCG patients with *CYP1B1* mutations is younger than in patients without *CYP1B1* mutations [34]. Our data show that the onset age in three patients (P60, P66, and P67) was 7, 3, and 11 months, while the rest of the patients presented at birth. In these 20 patients there is no significant difference in the age of disease onset in *CYP1B1* mutation-positive and mutation-negative cases, although clinical phenotypes of patients (P55, P56, P61, P68, and P70) with homozygous *CYP1B1* mutations were more severe compared to patients (P64 and P65) who were heterozygous for the *CYP1B1* mutations (Table 1). It is possible that patients with two null alleles with no catalytic activity may present with a more severe phenotype of the disease compared to patients with one null (heterozygote) allele. The disease phenotype of patients with homozygous/heterozygous *CYP1B1* mutations was more severe compared to the clinical phenotype of patients negative for the *CYP1B1* mutations.

We also observed a higher mean IOP in a group of patients with *CYP1B1* mutations. In accordance with the idea of associating the severe phenotypes with the null *CYP1B1* allele, the percentage of severe phenotypes in at least one eye has been reported to be associated with various mutations ranging 80-100% for a frameshift mutation (e.g., c.376insA) and truncating mutations [11]. Three different truncation mutations (p.C280X, p.E281X, and p.R355X) producing a truncated protein of 279, 280, and 354 amino acids, respectively, have also been associated with more severe disease phenotypes [11,21,29]. In patient P55 with a homozygous p.I94X mutation, a truncated protein of 93 amino acids is produced that has the first 82 amino acids similar to the wild-type CYP1B1 protein. The disease phenotype of this patient is severe with a left phthisic and a right buphthalmic eye with a cup to disc ratio of 0.9:1. He is visually blind. Another patient (P70) with a p.R355X mutation had bilateral buphthalmos with severe corneal edema and a corneal diameter of 15.0×15.0 mm and 15.0×14.5 mm in the left and right eye, respectively. He was blind at the age of 45 days. Patient P61 with a p.H279D mutation had bilateral buphthalmos with mild edema in both eyes and a corneal diameter of 15.5×15.5 mm and 14.0×14.0 mm in the left and right eye, respectively. He was blind at the age of 4 months. The range of percentages of severe phenotypes in at least one eye is 62–83% for different mutations, such as p.G61E, p.E229K, p.R368H, and p.R390C [9]. 

Membrane-bound cytochromes, such as CYP1B1, have a molecular structure containing a transmembrane domain located at the N-terminal end of the molecule. This is followed by a proline-rich “hinge” region, which permits flexibility between the membrane-spanning domain and the cytoplasmic portion of the protein molecule. The COOH-terminal ends are highly conserved among different members of the cytochrome P450 superfamily [17]. This family contains a set of conserved core structures responsible for the heme-binding region of these molecules. The heme-binding region is essential for the normal function of every P450 molecule. Between the hinge region and the conserved core structure lies a less conserved substrate-binding region. The cytochrome P450 protein functions like any classical enzyme molecule [18,19]. Mutations affecting such enzymes generally produce recessive phenotypes because in heterozygous subjects the normal allele is capable of compensating for the mutant allele. Mutations in the CYP1B1protein interfere with the integrity of the CYP1B1 protein as well as its ability to adopt a normal conformation and to bind heme; for example, induced mutations in the hinge region have previously been reported to interfere with the heme-binding properties of the cytochrome P450 molecules

Thus mutations of *CYP1B1* are a major cause of PCG in our study as well as various other studies [6-15,35-37]. This study confirms genetic heterogeneity of the disease. We identified four novel mutations in this study in addition to one previous reported [7]. Studies of pathogenic sequence variants of CYP1B1 in different populations will contribute to a better understanding of the pathogenesis of PCG and will aid in analyzing the structure–function relationship of different *CYP1B1* mutations Identifying mutations in subjects at risk of developing glaucoma, particularly among relatives of PCG patients, is of clinical significance. Monitoring vision in these families would be helpful. These developments may help in reducing the disease frequency in familial cases. Such studies will also help in understanding the pathogenic mutations in our patient populations and enable us to develop simple and rapid diagnostic tests for analyzing such cases. This may lead to the development of novel therapies in the management of congenital glaucoma.
